# S-adenosyl-L-homocysteine hydrolase FgSah1 is required for fungal development and virulence in *Fusarium graminearum*

**DOI:** 10.1080/21505594.2021.1965821

**Published:** 2021-08-23

**Authors:** Dongya Shi, Yu Zhang, Jin Wang, Weichao Ren, Jie Zhang, Jane Ifunanya Mbadianya, Yuanye Zhu, Changjun Chen, Hongyu Ma

**Affiliations:** a Department of Pesticide Science, College of Plant Protection, Nanjing Agricultural University, Nanjing, China; b Department of Crop Protection, Zhejiang Agriculture and Forest University, Hangzhou, China; c Department of Plant Pathology, College of Plant Health and Medicine, Qingdao Agricultural University, Qingdao, China

**Keywords:** *Fusarium graminearum*, FgSah1, methylation metabolism, vegetative growth, stress responses, lipid metabolism

## Abstract

The S-adenosyl-L-homocysteine hydrolase (Sah1) plays a crucial role in methylation and lipid metabolism in yeast and mammals, yet its function remains elusive in filamentous fungi. In this study, we characterized Sah1 in the phytopathogenic fungus *F. graminearum* by generating knockout and knockout-complemented strains of *FgSAH1*. We found that the FgSah1-GFP fusion protein was localized to the cytoplasm, and that deletion of *FgSAH1* resulted in defects in vegetative growth, asexual and sexual reproduction, stress responses, virulence, lipid metabolism, and tolerance against fungicides. Moreover, the accumulations of S-adenosyl-L-homocysteine (AdoHcy) and S-adenosyl-L-methionine (AdoMet) (the methyl group donor in most methyl transfer reactions) in Δ*FgSah1* were seven- and ninefold higher than those in the wild-type strain, respectively. All of these defective phenotypes in Δ*FgSah1* mutants were rescued by target gene complementation. Taken together, these results demonstrate that *FgSah1* plays essential roles in methylation metabolism, fungal development, full virulence, multiple stress responses, lipid metabolism, and fungicide sensitivity in *F. graminearum*. To our knowledge, this is the first report on the systematic functional characterization of Sah1 in *F. graminearum*.

## Introduction

The ascomycete fungus *Fusarium graminearum* (teleomorph: *Gibberella zeae*) is a predominant causal agent of Fusarium head blight (FHB), which is a disastrous disease on various cereal crops [1,2]. Severe FHB epidemics result in devastating yield loss and the contamination of infected grains with mycotoxins produced by *F. graminearum* (e.g. deoxynivalenol (DON), zearalenone), which are toxic to humans and livestock [2,3]. To date, no commercially FHB-resistant cultivars are available, and fungicides are heavily relied upon to control infestations worldwide. However, the extensive use of chemical control has negative effects, including the resistance development against fungicide [2,4,5]. Therefore, a better understanding of the mechanisms underlying the procedure of pathogenicity in *F. graminearum* is essential to develop better target-based control strategies [6].

**Figure 1.**
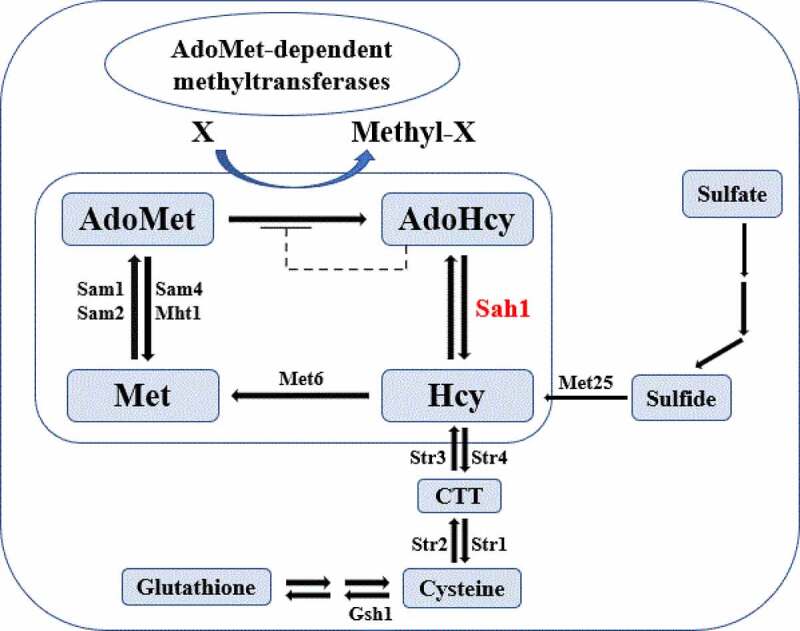
Model of the methionine cycle in yeast. Sah1 is marked in a red font

The methionine cycle is a pathway well documented in yeast and mammals. As summarized in Figure 1, methionine is first catalyzed into S-adenosyl-L-methionine (AdoMet) by methionine adenosyltransferase, then AdoMet is converted into S-adenosyl-L-homocysteine (AdoHcy) by the removal of a methyl group, subquently AdoHcy is hydrolyzed by Sah1 into homocysteine (Hcy), and methionine is regenerated from Hcy when catalyzed by methionine synthase (Met6) [7,8]. This cycle is tightly linked to various other cellular processes, as AdoMet is the second most widely used enzyme substrate after ATP [9], functioning as the major methyl group donor for AdoMet-dependent methyltransferases reactions, in which acceptor molecules range from nucleic acids to proteins and lipids [8]. At the same time, AdoHcy, which is a by-product of methylation reaction, is a strong competitive inhibitor of all AdoMet-dependent methyltransferases [10]. To relieve AdoHcy inhibition, AdoHcy is reversibly hydrolyzed into adenosine and Hcy by Sah1, which is the singular pathway of AdoHcy catabolism both in yeast and mammals (Sah1 in yeast, AHCY in mammals) [8]. Because the reaction is reversible and there are no other enzymes capable of hydrolyzing AdoHcy, an increase of Hcy levels may induce the formation of AdoHcy and the resulting accumulation may lead to feedback inhibition in the biosynthesis of AdoHcy from AdoMet.

The S-adenosyl-L-homocysteine hydrolase (Sah1) is a protein well conserved from bacteria to mammals with more than 70% amino acid sequence homology between human and yeast orthologs and also among the 90 most highly conserved yeast proteins [11]. Impaired Sah1 activity in most living organisms results in AdoHcy accumulation and severe pathological consequences. For example, mouse embryos cannot survive after deletion of a locus overlapping the *AHCY* gene [12], and homozygous mutations in the zygotic *SAH1* gene were observed to be lethal to *Arabidopsis thaliana* [13]. Although yeast mutants without Sah1 are viable due to the presence of an alternative pathway for Hcy synthesis via sulfur assimilation, additional mutation in this pathway is lethal for the resulting yeast *sah1* mutants [14]. In addition to its integral role in virtually all living organisms, Sah1 is also known to belong to the large family of NAD(P)H/NAD(P)^+^-binding proteins with a Rossmann-fold [15]. The structures of Sah1/AHCY from various organisms have been characterized [8], of which only the plant Sah1 from *Lupinus luteus* works as a homodimer [16], while others are tetramers with the NADH/NAD^+^ cofactor bound at active sites of each subunit [17,18].

Owing to its central role in methionine cycle, Sah1 may be a potential target for fungicide interest. In this study, we identified and characterized Sah1 in *F. graminearum* (FgSah1). Our results indicate that FgSah1 plays diverse roles in *F. graminearum*, including methylation metabolism, vegetative growth, asexual and sexual reproduction, stress responses, DON production, full virulence, and lipid metabolism.

## Materials and methods

### Fungal strains and media

The wild-type (WT) strain of *F. graminearum* PH-1 and transformants derived from PH-1 were used in this study. All strains were routinely incubated on PDA and CM plates for mycelial growth test [19]. Liquid YEPD (w/v, 1% peptone, 0.3% yeast extract, 2% dextrose) was used to prepare mycelia for the extraction of genomic DNA and total RNA. Mung bean liquid (MBL) broth (30 g mung beans boiled in 1 L water for 20 min, then filtered through cheesecloth) was used to analyze induction of asexual reproduction. Water agar medium was used for conidia germination experiments [20]. Carrot agar (CA) medium was used for self-crossing assays. Potato dextrose broth (PDB) was used for pigmentation production and total RNA extraction. Each experiment was repeated at least three times.

### Gene deletion and complementation by protoplast transformation

The gene deletion mutants in the PH-1 background were generated with a homologous recombination strategy (Figure S1A), and the gene replacement cassette (*HPH*) was constructed with a double-joint PCR approach [21]. Protoplast preparation and transformation were performed following previously described methods [20]. The deletion mutants identified from hygromycin-resistant (100 µg/mL) transformants were verified by diagnostic PCR and further confirmed by Southern blotting (Figure S1B). For complementation assays, the native promoter and open-reading frame of *FgSAH1* were amplified from the *F. graminearum* genome and co-transformed with pYF11 vector (neomycin-resistance marker NEO) harboring green fluorescent protein (GFP) into *E. coli* using described methods [22]. The resulting pYF11 plasmids were transformed into protoplasts of the Δ*FgSah1* mutant and the candidate transformants resistant to G418 (geneticin) at 100 µg/mL were screened with PCR and fluorescence signals. All primers used for these experiments are listed in Supplementary Table S1.

### Quantification of AdoMet and AdoHcy production

The mycelia grown in YEPD for 2 d were harvested to measure the content of AdoMet and AdoHcy with HPLC procedures by Poirier et al. [23] with minor modifications. Briefly, mycelial powder (1 g) grinded under liquid nitrogen was added to a 10 mL centrifuge tube. The tube was filled to 5 mL with ultrapure water, then underwent 10 minutes of homogenization and 5 minutes of ultrasonic vibration. The mixture was centrifugated at 13,000 rpm for 20 min, then the supernatant was aspirated, after which the experimental protocol followed the methods of Poirier [23].

### Asexual and sexual reproduction assays

For asexual reproduction assays, five mycelial plugs (5 mm in diameter) taken from the edge of a 3-d-old colony of each strain were added to a 50-mL triangular flask containing 30 mL of MBL broth. Each strain was assayed with three flasks and incubated at 25°C for 5 d with shaking (175 rpm). The number of conidia in each flask was calculated using methods by Zheng et al. [24] and the experiment was repeated three times. In conidia germination experiments, the conidia of PH-1, Δ*FgSah1* and Δ*FgSah1-C* were coated evenly on thin layer of water agar plates and incubated at 25°C for 8 h, then examined with an Olympus IX-71 microscope (Tokyo, Japan).

For self-crossing assays, 7-d-old aerial hyphae on carrot agar (CA) cultures were gently removed with a sterile toothpick with the addition of 1 mL autoclaved 2.5% Tween 20 following previously described protocol [25,26]. After additional 2 weeks of incubation at 25°C under a 12 h of light (bright fluorescent lights) −12 h of darkness diurnal cycle, perithecia produced on CA plates were observed and photographed using a stereo microscope (Nikon SMZ25). All the experiments were performed three times with three independent replicates.

### Plant infection, penetration, and DON production assays

To test the pathogenicity of Δ*FgSah1* on wheat coleoptiles and flowering wheat heads, virulence assays were performed as previously described [27]. Briefly, for coleoptile pathogenicity, 2–3 mm sections from the top of 3-d-old seedlings were removed and the wound was inoculated with a 2.5-μL aliquot conidial suspension (3 × 10^5^ spores/mL) of each strain. For wheat ear pathogenicity, a 10-μL aliquot of conidial suspension (3 × 10^5^ spores/mL) was point-inoculated into a floret of flowering wheat head of Zhenmai 5, and sterile distilled water was used as negative control. More than 20 plants were inoculated with each strain, and disease symptoms were determined at 7 (coleoptiles) or 14 (wheat heads) d post inoculation (dpi). In addition, cellophane penetration assays of Δ*FgSah1* mutants were tested using methods described before [28]. Briefly, mycelial plugs (5 mm in diameter) on cellophane membranes placed on CM plates were cultured at 25°C for 3 d in the dark, then fungal colonies from each strain were removed with the cellophane membranes, and the plates were incubated for another 2 d under the same condition to assess the penetration of the mycelia.

DON production was assayed as described previously [28,29]. Briefly, healthy wheat grains soaked in sterile water for 24 h were divided into 250-mL triangular flasks (a 50-g aliquot) and autoclaved for 20 min twice. After being cooled to room temperature, each treatment was inoculated with 1 mL of spore suspension (3 × 10^5^/mL) and shaken at intervals of 12 h to ensure even infection. After the mixture was incubated at 25°C for 20 d in the dark, DON production was determined using a DON ELISA Assay Kit (Wise, Zhenjiang, 465 China) according to methods described before [29,30]. The amount of fungal ergosterol in each sample was estimated and used as the internal control [31]. The experiment was repeated three times with three replicates independently.

### Microscopic examinations

Hyphal and conidial morphology were observed with an inverted microscope (Zeiss LSM710/780 Germany). The mycelia of each strain originated from the mycelial plugs which were cultured on cellophane membranes placed on solidified PDA plates for 2 d, and the conidia were harvested from MBL cultures after 5 d of cultivation. To determine spore morphology and septum number, conidia of each strain were stained with 10 µg/mL calcofluor white (CFW) and examined with a confocal laser scanning microscope (Leica TCS SP8, Germany). Lipid droplets (LDs) in mycelia were observed by staining with Nile Red as described before [32].

### Sensitivity of mycelial growth to multiple stresses

To test the sensitivity of Δ*FgSah1* mutant to multiple stresses, mycelial growth was assayed on PDA plates without or with 1.2 M NaCl or KCl, 0.05% SDS (w/v), 0.05% Congo red (w/v), 8 mM hydrogen peroxide (H_2_O_2_). To test the sensitivity of Δ*FgSah1* mutant against different fungicides, mycelial plugs (5 mm in diameter) taken from the fringe of a 3-d-old colony of each strain were placed on the center of PDA plates amended with fludioxonil (0, 0.003125, 0.00625, 0.0125, 0.025, 0.05, 0.1, and 0.2 µg/mL) or iprodione (0, 0.5, 1, 2, 4, 8, 16, and 32 µg/mL). The plates were cultivated under 25°C for 3 d in the dark, and all experiments were repeated three times independently.

### Investigation of intracellular glycerol content

Two-d-old mycelia of each strain were harvested, and 100 mg samples of mycelia were ground under liquid nitrogen. The glycerol concentrations in mycelia were determined with a Glycerol Assay Kit (Applygen Technologies Inc., Beijing, China). The experiments were repeated three times independently.

### Protein manipulation and western blotting

Protein extraction was performed using protocols described previously [33]. The concentrations of the total proteins were measured with a Bradford Protein Assay Kit (Beijing Solarbio Science & Technology Co., Ltd.), and 0.05 mg of each sample was separated by 12% sodium dodecyl sulfate polyacrylamide gel electrophoresis (SDS-PAGE). The proteins were further transferred to Immobilon-P transfer membranes (Millipore, Billerica, USA), then phosphorylation of FgHog1 and total FgHog1 were detected by Phospho-p38 MAPK (Thr180/Tyr182) Antibody #9211 (Cell Signaling Technology Inc., Boston, MA, USA) and anti-Hog1 antibody sc165978 (Santa Cruz, CA, USA), respectively. Protein abundance was quantified with ImageJ software. The experiments were performed three times independently.

### Quantitative real-time PCR (qRT‑PCR)

Total RNAs of *F. graminearum* mycelia harvested from YEPD, PDB, cellophane layers placed on CM plates, and DON induction medium were extracted with the RNAiso reagent (Tiangen Biotech, Beijing, China). First-strand cDNA was synthesized with a HiScript® II 1st Strand cDNA Synthesis Kit* (Vazyme Biotech, Nanjing, China). The actin gene was used as the internal control, and the relative gene expression levels were evaluated by the threshold cycle (2^−ΔΔCt^) method [34]. The primers used in this study are presented in Table S1.

### Statistical analyses

All the experimental data were analyzed with the Date Processing System (DPS, version 7.05), and the means from triplicate samples were analyzed by Fisher’s least significant difference (LSD) test. Each error bar represents the standard deviation (SD) of three independent experiments.

## Results

### *Identification of* FgSAH1 *in* F. graminearum

**Figure 2.**
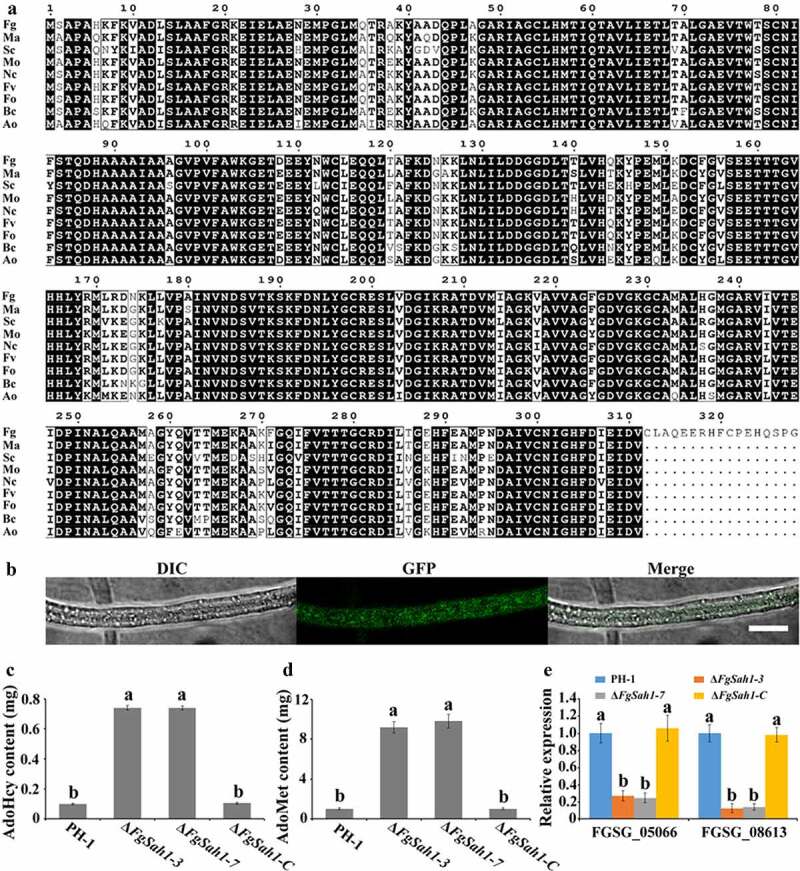
Identification of FgSah1 in *F. graminearum*

A hydrolase gene (FGSG_05615, designated FgSah1) was retrieved with a BLASTp search of the NCBI database using *S. cerevisiae* Sah1 (YER043 C) as a query. The *FgSAH1* gene is 987 bp and encodes a 328 amino acid protein. Both homologous alignment (Figure 2(a)) and phylogenetic analyses (Figure S2) which were based on the amino acid sequences indicated that the Sah1 protein of *F. graminearum* is evolutionarily conserved.

(a) Sequence alignment of FgSah1 with other fungal Sah1. Fg, *Fusarium graminearum*; Ma, *Metarhizium anisopliae*; Sc, *Saccharomyces cerevisiae*; Mo, *Magnaporthe oryzae*; Nc, *Neurospora crassa*; Fv, *Fusarium verticillioides*; Fo, *Fusarium oxysporum*; Bc, *Botrytise cinerea*; Ao, *Aspergillus oryzae*. The black background indicates the same amino acid residues. (b) Subcellular localization of the FgSah1-GFP fusion protein in *F. graminearum*. Bar = 10 µm. (c) AdoHcy content in the wild-type strain PH-1, the deletion mutants Δ*FgSah1-3* and Δ*FgSah1-7*, and the complemented mutants Δ*FgSah1-C*. (d) AdoMet accumulation in the tested strains. (e) The expression levels of two AdoMet-dependent methyltransferases. Means and standard deviations (SDs) were calculated from three independent experiments. Values on the bars followed by different letters are statistically different according to the Fisher’s least significant difference (LSD) test at *p* < 0.05.

The identified Sah1 was primarily detected in the cytosol in yeast and found to localize to the nucleus and cytoplasm in *A. thaliana* [8]. The signal of green fluorescence protein (GFP), tagged at the C terminus of FgSah1 to visualize its subcellular localization in *F. graminearum*, was observed to distribute evenly in the hyphae (Figure 2(b)), suggesting that FgSah1 is localized to the cytoplasm.

### *AdoMet-dependent methylation metabolism is blocked in Δ*FgSah1

As the second most widely used enzyme substrate, AdoMet provides methyl groups for most methylation reactions in all organisms. In this process, it is converted into its demethylated metabolite AdoHcy, whose accumulation can work as the strong competitive inhibitor of all AdoMet-dependent methyltransferases [8,35]. To investigate whether FgSah1 is responsible for catabolizing AdoHcy in *F. graminearum*, the intracellular content of AdoMet and AdoHcy was determined, and results showed that the *FgSAH1* deletion mutants produced sevenfold more AdoHcy and ninefold more AdoMet than those of WT PH-1 and the complemented strain Δ*FgSah1-C* (Figure 2(c,d)). This strongly suggests that the deletion of *FgSAH1* leads to the accumulation of AdoHcy, which negatively affects the conversion of AdoMet to AdoHcy and then causes the accumulation of AdoMet as well (Figure 1). It has also been reported that *de novo* biosynthesis of phosphatidylcholine (PC) from phosphatidylethanolamine (PE) is based on three sequential AdoMet-dependent methylation steps catalyzed by Cho2 (Phosphatidylethanolamine N-methyltransferase) and Opi3 (bifunctional phosphatidyl-N-methylethanolamine N-methyltransferase/phosphatidyl-N-dimethylethanolamine N-methyltransferase) both in yeast and in mammals [8,36]. In this study, we investigated the expression levels of Cho2 and an Opi3-like gene (FGSG_05066 and FGSG_08613) in Δ*FgSah1*, finding that the knockout mutants showed dramatically decreased mRNA expression levels of these two AdoMet-dependent methyltransferases (Figure 2(e)). All results demonstrate that FgSah1 is essential to methylation in *F. graminearum*.

### *FgSah1 is involved in vegetative growth and pigmentation in* F. graminearum

**Figure 3.**
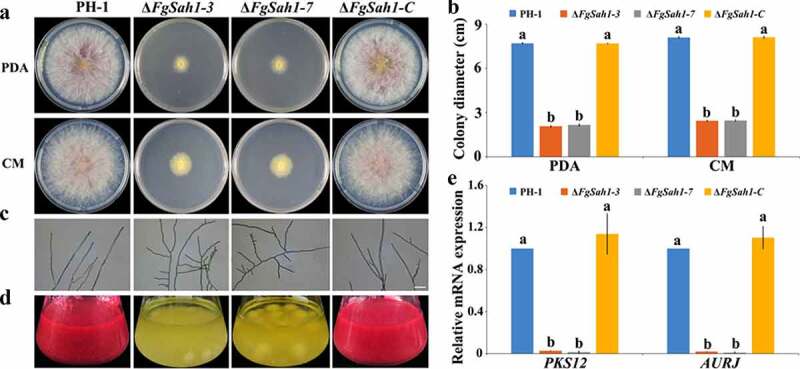
Roles of FgSah1 in vegetative growth and pigmentation in *F. graminearum*

As shown in Figure 3(a,b), the colonial growth rates of the *FgSAH1* deletion mutants decreased dramatically compared to PH-1 and Δ*FgSah1-C* on PDA and CM media, and in contrast to the control strains, which produced abundant aerial hyphae, the Δ*FgSah1* mutants produced fewer and shorter aerial hyphae. Moreover, microscopic examination showed that most hyphal branches of the deletion mutants had larger branching angles than those of PH-1 and Δ*FgSah1-C* (Figure 3(c)). Furthermore, after incubated in PDB for 5 ds, the Δ*FgSah1* mutants produced yellow instead of red pigments (Figure 3(d)). After 2 weeks, the Δ*FgSah1* mutants still failed to produce any red pigments in PDB medium, and the same results were observed on PDA and CM plates (date not shown). To further confirm this phenomenon, 2-d-old mycelia in PDB were harvested for RNA extraction, and relative gene expression levels of *PKS12* and *AURJ*, known to be responsible for the synthesis of the red pigment aurofusarin [37], were determined by qRT-PCR analyses. The results showed that the mRNA expression levels of the two genes were significantly down-regulated in the Δ*FgSah1-3* and Δ*FgSah1-7* mutants compared to PH-1 and Δ*FgSah1-C* (Figure 3(e)), indicating that FgSah1 plays a crucial role in pigment formation in *F. graminearum*.

(a) Colony morphology of the wild-type strain PH-1, the deletion mutants Δ*FgSah1-3* and Δ*FgSah1-7*, and the complemented strain Δ*FgSah1-C* on PDA, CM media at 25°C for 3 d. (b) Colony diameters measured in each independent biological experiment and statistically analyzed by LSD test. Bars represent standard deviations from three replications. (c) Hyphal tip morphology and branching patterns of PH-1, Δ*FgSah1-3*, Δ*FgSah1-7*, and Δ*FgSah1-C* on water agar. Bar = 50 µm. (d) Liquid culture in flasks containing 150 mL of PDB medium at 25°C for 5 d. (e) Relative mRNA expression levels of aurofusarin biosynthesis-related genes *FgPKS12* (FGSG_12040), *FgAURJ* (FGSG_02326). The Actin gene was used as an internal control. Means and SDs were calculated from three independent experiments. Different letters indicate statistically significant differences at *P* < 0.05.

### FgSah1 is important for both asexual and sexual reproduction

**Figure 4.**
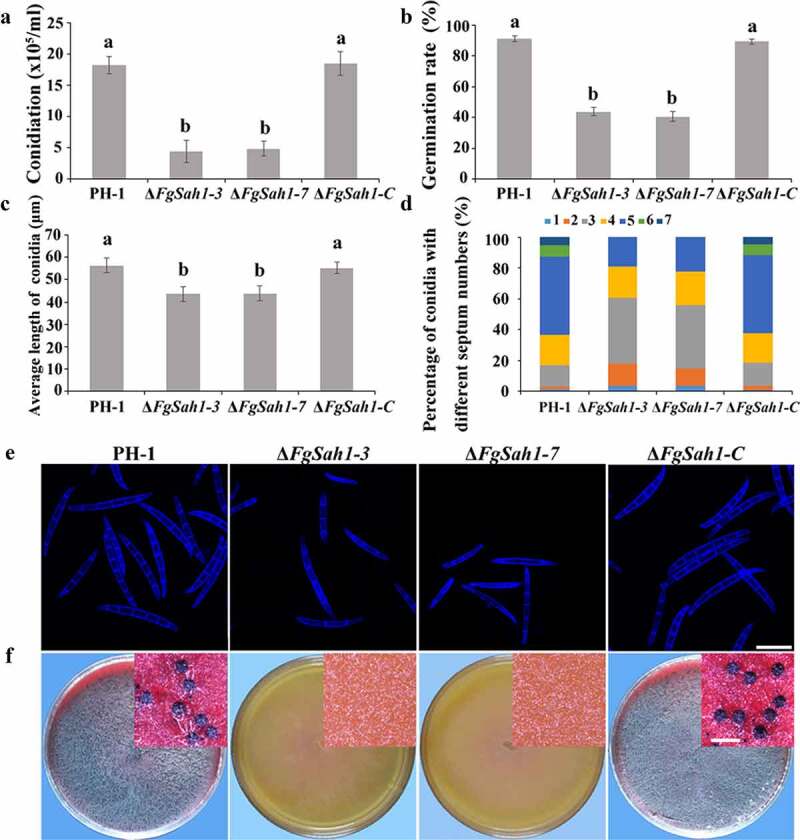
Assays for asexual and sexual reproduction in the *FgSAH1* deletion mutants

To investigate whether FgSah1 is involved in asexual development, the spore morphology and conidial production of PH-1, Δ*FgSah1*, and Δ*FgSah1-C* strains were examined in MBL medium. The number of conidia produced by Δ*FgSah1* was significantly lower (about 70% less) compared to that of PH-1 and Δ*FgSah1-C* strains (Figure 4(a)), and the conidial germination rates (for 8 h on water agar at 25°C) were also dramatically decreased (Figure 4(b)). Microscopic observations also revealed that not only was the length of conidia produced by deletion mutants reduced (Figure 4(c)), but the number of septa of the conidia also declined in Δ*FgSah1* in comparison to PH-1 and Δ*FgSah1-C* (Figure 4(d,e)). Apart from asexual reproduction, the sexual reproduction of Δ*FgSah1* was assessed on carrot agar plates. After 2 weeks of incubation, the deletion mutants were sterile while the wild-type strain PH-1 and complemented strain Δ*FgSah1-C* produced abundant perithecia (Figure 4(f)). The above results suggest that FgSah1 is crucial to both asexual and sexual reproduction, and thus may be a potential fungicide target for controlling the spread of FHB.

(a) Amount of conidia produced by the wild-type strain PH-1, the deletion mutants Δ*FgSah1-3* and Δ*FgSah1-7*, and the complemented strain Δ*FgSah1-C*. (b) Germination rate of the conidia produced by PH-1, Δ*FgSah1-3*, Δ*FgSah1-7* and Δ*FgSah1-C* on water agar plates at 25°C for 8 h. (c) Lengths of conidia (n = 400) produced by each strain. (d) Percentage of spores in each strain containing 0–7 septa (n = 200). (e) Septa of conidia produced by each strain visualized after staining with calcofluor white (CFW); bar = 25 μm. (f) Perithecia produced by each strain; bar = 500 μm. Means and SDs were calculated from three independent experiments. Different letters indicate statistically significant differences at *P* < 0.05.

### FgSah1 is required for full virulence

**Figure 5.**
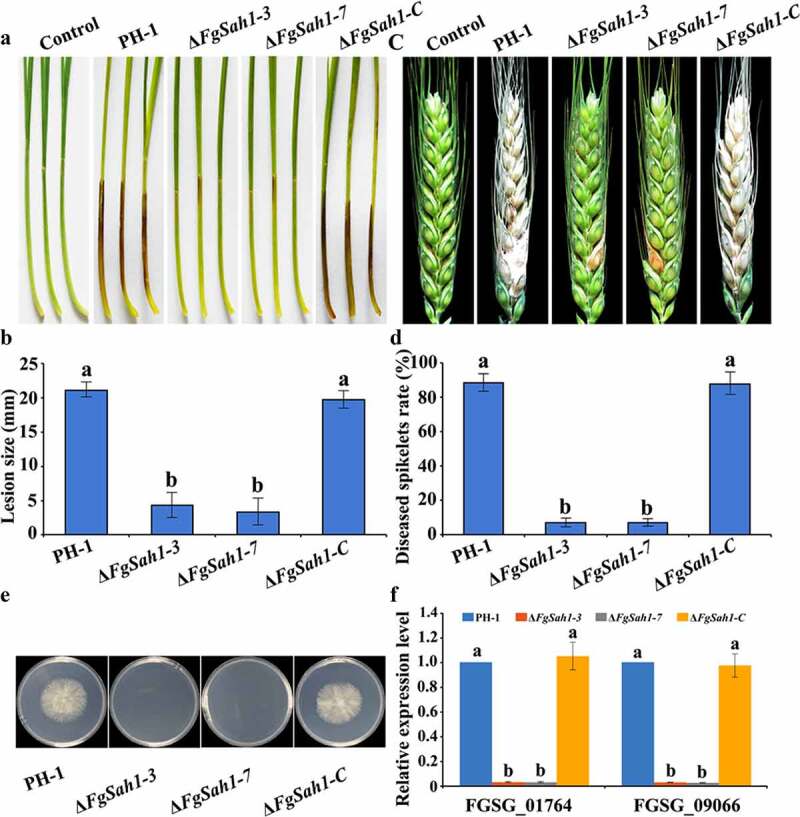
Pathogenicity and cellophane penetration assays of the *FgSAH1* deletion mutants

To test whether the *FgSAH1* gene is involved in the pathogenicity of *F. graminearum*, infection tests were performed on wheat coleoptiles and flowering wheat heads. The deletion mutants of *FgSAH1* exhibited reduced virulence and infections were limited to the wound of coleoptiles or the inoculated spikelet, whereas symptoms on coleoptiles and flowering wheat heads of PH-1 and Δ*FgSah1-C* were typically severe (Figure 5(a,d)). Previous studies reported that loss of virulence may be a result from defective penetration ability of the mutant strains [38,39]. This is affirmed in the present study, where cellophane penetration assays demonstrated that the deletion mutants failed to penetrate cellophane membranes (Figure 5(e)); even when incubated for an additional 2 weeks, the plates of deletion mutants produced no hyphae or colonies. This result suggests that FgSah1 plays important roles in penetration during infection. Apart from this, the mRNA expression levels of hydrophobins (FGSG_01764 and FGSG_09066), which have been reported to be involved in cellophane penetration and plant infection [40,41], were determined in all tested strains, and results showed that the relative expressions of the two hydrophobins in Δ*FgSah1* were greatly down-regulated (Figure 5(f)). Disease assays on coleoptiles performed by spray inoculations were further confirmed the mutant loses the ability to infect healthy plants, suggesting that FgSah1 affects *F. graminearum* penetration during plant infection (Figure S3). All these results indicate that FgSah1 is essential to full virulence in *F. graminearum*.

(a) Wheat coleoptiles were point-inoculated with conidial suspensions and examined 7 d post-inoculation (dpi). Sterile distilled water was used as the control. (b) Lesion size of the infested coleoptiles. (c) Flowering wheat heads were inoculated with conidial suspensions and examined 14 dpi. (d) Percentage of diseased spikelets on the infected wheat heads. (e) The Δ*FgSah1* mutants were unable to penetrate cellophane membranes. (f) The mRNA expression levels of the two hydrophobins. Error bar indicates the standard deviation (SD) calculated from three independent experiments, values on the bars followed by different letters are statistically different at *p* < 0.05.

**Figure 6.**
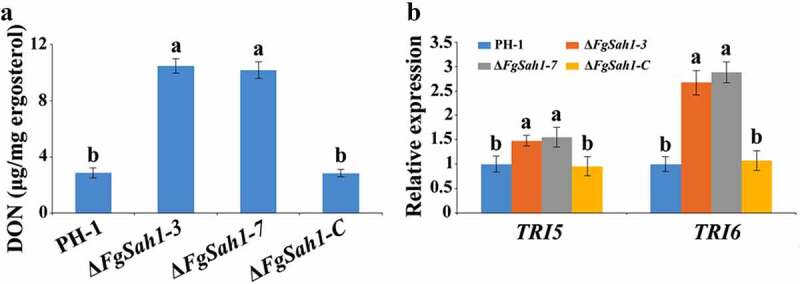
DON production and *TRI* genes expression assays of the *FgSAH1* deletion mutants

As a key pathogenic factor of *F. graminearum* [42,43], DON production was assessed in Δ*FgSah1* infecting wheat grains. In contrast to tests on pathogenicity, DON production in Δ*FgSah1* was significantly increased (Figure 6(a)). To further confirm this result, the relative expression levels of DON biosynthesis genes *TRI5* and *TRI6* in minimal synthetic (MS) liquid medium [28] were analyzed by qRT‑PCR, and the results showed that expressions of *TRI5* and *TRI6* were significantly up-regulated (Figure 6(b)). All these results indicate that FgSah1 is required for full virulence but also functions redundantly to negatively regulate DON production.

(a) DON concentration (μg/mg fungal ergosterol) in infected wheat kernels produced by each strain assayed after 20 d of inoculation. (b) Relative mRNA expression levels of DON biosynthetic genes *TRI5* and *TRI6* in each strain. After cultured in MS liquid medium for 4 d at 25°C in the dark, the mycelia of each strain was harvested and each RNA sample was extracted. Means and SDs were calculated from three independent experiments. Values on the bars followed by different letters are statistically different at *p* < 0.05.

### Sensitivity of the FgSAH1 deletion mutants to multiple stresses

**Figure 7.**
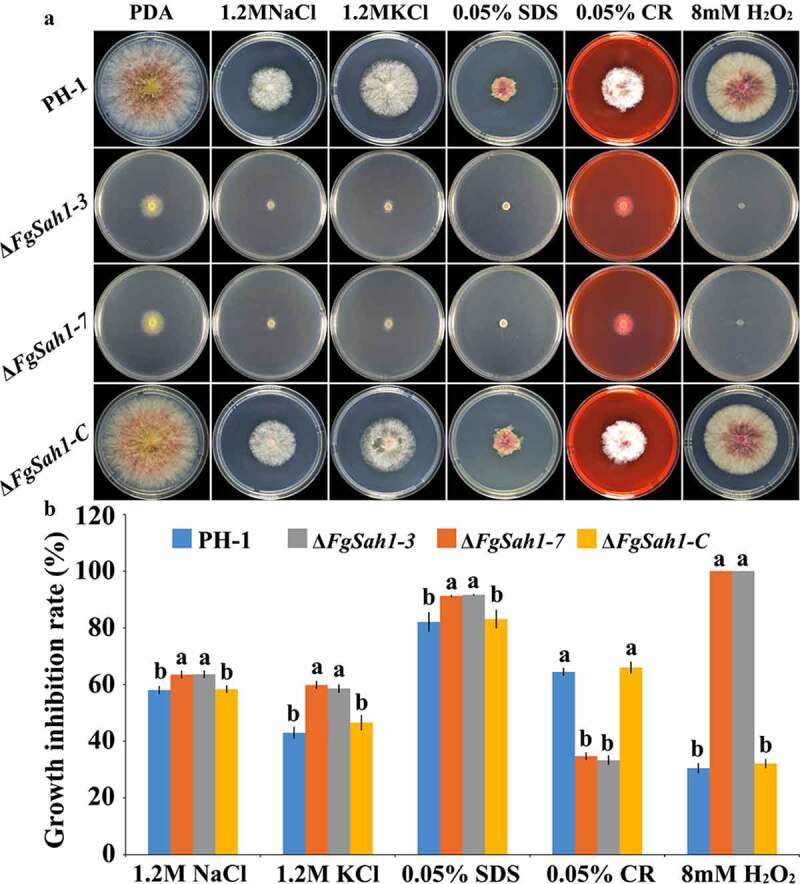
Sensitivity of the *FgSAH1* deletion mutants to osmotic, membrane, cell wall damage, and oxidative stresses

Full virulence of *F. graminearum* has been reported to be impaired by the depletion of genes involved in various stress responses, including osmotic, cell membrane, cell-wall related, and oxidative stresses [22,44]. Tolerance to environmental stresses is critical for plant infection, and thus the response of Δ*FgSah1* to various stressors was examined. The *FgSAH1* deletion mutants, wild-type strain PH-1, and the complemented strain Δ*FgSah1-C* were inoculated on PDA plates with each of 1.2 M NaCl or KCl (osmotic stresses), 0.05% SDS (membrane stress), 0.05% CR (cell wall stress), and 8 mM H_2_O_2_ (oxidative stresses). Intriguingly, the mycelia of Δ*FgSah1* showed increased sensitivity to NaCl, KCl, SDS, and H_2_O_2_, but decreased sensitivity to CR compared to those of PH-1 and Δ*FgSah1-C* (Figure 7(a,b)). These results demonstrate that the hydrolase FgSah1 is also critical in the response to abiotic stresses in *F. graminearum*.

(a) The wild-type strain PH-1, the deletion mutants Δ*FgSah1-3* and Δ*FgSah1-7*, and the complemented strain Δ*FgSah1-C* cultured on PDA medium amended without or with 1.2 M NaCl, 1.2 M KCl, 0.05% (w/v) SDS, 0.05% (w/v) Congo red (CR) and 8 mM H_2_O_2_. (b) Growth inhibition rate of each strain after incubation at 25°C for 3 d. Means and SDs were calculated from three independent experiments. Values on the bars followed by different letters are statistically different at *p* < 0.05.

### FgSah1 is involved in lipid metabolism

**Figure 8.**
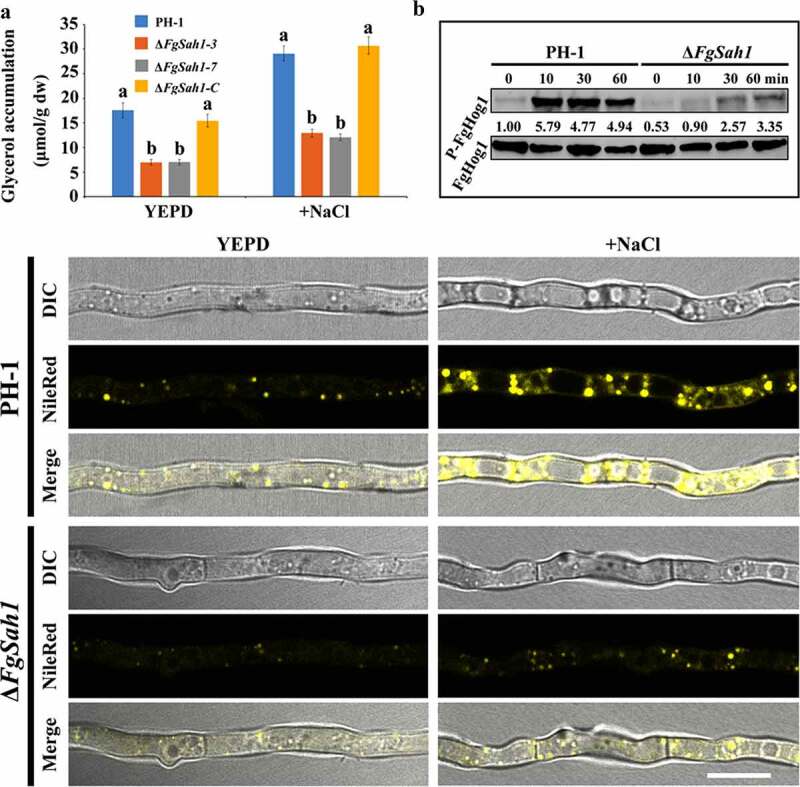
*FgSAH1* is involved in lipid metabolism in *F. graminearum*

Normally, external hyperosmotic stresses activate the high-osmolarity glycerol (HOG) pathway through which the intracellular glycerol content is regulated by Hog1 phosphorylation [45,46]. In this study, glycerol content and the phosphorylation of FgHog1 were examined. The results demonstrate that both under normal and hyperosmotic conditions, the deletion mutants exhibited decreased glycerol accumulation than PH-1 and Δ*FgSah1-C* (Figure 8(a)). In accordance with this result, the phosphorylation levels of FgHog1 in Δ*FgSah1* were dramatically decreased compared to that of the wild-type strain PH-1 (Figure 8(b)). These results indicate that FgSah1 plays a significant role in regulating the activity of FgHog1 and the biosynthesis of glycerol, and in turn maintains osmotic homeostasis in *F. graminearum* in response to external osmotic stresses.

(a) Intracellular glycerol accumulation in PH-1, Δ*FgSah1*, and Δ*FgSah1-C* with or without NaCl (1.2 M) treatment for 2 hours. (b) The total and phosphorylated FgHog1 detected by p38 MAP and phosphor-p38 MAP kinase antibodies, respectively. (c) Nile red used to stain lipid droplets (LDs) in hyphae of each strain after treatment with or without 1.2 M NaCl for 2 h; bar = 10 μm.

Since glycerol has been reported to be the raw material for LD biosynthesis [47,48], the accumulation of glycerol in Δ*FgSah1* can be further confirmed by the formation of LDs. Mycelia of each strain were stained using Nile Red, a selective fluorescent stain for intracellular LDs [49], and observed by fluorescence microscopy. As shown in Figure 8(c), the *FgSAH1* deletion mutant exhibited fewer LDs than those of PH-1 even under 1.2 M NaCl (2 h). All these results indicate that FgSah1 is required for cellular lipid homeostasis in *F. graminearum*.

### *Sensitivity of Δ*FgSah1 *to different fungicides*

The HOG pathway has also been reported to be related to the sensitivity toward phenylpyrrole and dicarboximide fungicides in some fungal pathogens [50]. Since our results above showed that the decrease of phosphorylation level of FgHog1 affected the accumulation of glycerol in Δ*FgSah1*, we speculate that FgSah1 is involved in the sensitivity of *F. graminearum* to fludioxonil and iprodione. To test this hypothesis, the effective concentration for 50% inhibition of mycelial growth (EC_50_) of fludioxonil and iprodione for each strain was tested, and results showed that the deletion mutants exhibited increased sensitivity to fludioxonil and iprodione than PH-1 and Δ*FgSah1-C* (Table 1), which further confirmed the involvement of FgSah1 in the accumulation of intracellular glycerol in *F. graminearum*.

**Table 1.** Sensitivity of Δ*FgSah1* to different fungicides

Strain	EC_50_ (μg/mL)*	
	Fludioxonil	Iprodione
PH-1	0.0413a	8.2212a
∆*FgSah1-3*	0.0132b	5.1919b
∆*FgSah1-7*	0.0127b	5.1239b
∆*FgSah1-C*	0.0397a	7.9577a

* Means and standard deviations were calculated from three independent experiments. Means in the columns followed by the same letter are not significantly different according to the Fisher’s least significant difference (LSD) test at *P* < 0.05.

## Discussion

Methionine cycle is essential to growth and development of nearly all living organisms, and its dysfunction can cause various diseases and mortality [7,12–14]. In this study, we investigated the function of *FgSAH1* which encodes the S-adenosyl-L-homocysteine hydrolase (Sah1). Knowing that the dysfunction of Sah1 is lethal in many organisms including mice and *A. thaliana* [8], the survival of *FgSAH1* deletion mutants indicates that an alternative pathway for Hcy synthesis may be present in *F. graminearum*, which could be similar to that in yeast (Figure 1). In the disruption of FgSah1, the contents of AdoMet and AdoHcy in Δ*FgSah1* mutants were significantly increased and the expression levels of AdoMet-dependent methyltransferases were remarkably down-regulated, consistent with that in yeast and mammals. The accumulation of AdoHcy functions as a negative regulatory factor to the conversion of AdoMet, to the extent that AdoMet-dependent methylation processes were significantly inhibited. In *FgSAH1* deletion mutants, the accumulation of LDs was also decreased drastically, which indicates that FgSah1 is involved in lipid metabolism. All the above results indicate that FgSah1 plays crucial roles in methylation and lipid metabolism.

FgSah1 is essential to methylation metabolism in *F. graminearum*. Previous studies have demonstrated that only a few AdoMet-dependent methyltransferases are unique to yeast or humans while most are strongly conserved [8]. In this study, we found that the expression levels of the two AdoMet-dependent methyltransferases orthologs (Cho2 and Opi3 in yeast) in *F. graminearum* were dramatically inhibited (Figure 2(e)). Consistent with reports in yeast, the lack of FgSah1 resulted in massive accumulation of AdoHcy and AdoMet (Figure 2(c,d)), which disrupted the coordinated methylation processes. These results demonstrate that FgSah1 is involved in the regulation of methylation metabolism in *F. graminearum.*

FgSah1 is also required for vegetative growth and full virulence in *F. graminearum*. In *FgSAH1* deletion mutants, colony growth rate decreased significantly, and abnormal branching angles were observed in mycelia. The mutants also failed to produce the red pigment aurofusarin, an important secondary metabolite [51]. Previous studies reported that the synthesis of aurofusarin is dependent on the *PKS12* gene cluster [52–54], whose primary product is nor-rubrofusarin (a new yellow/green compound) [37]. This product is further catalyzed by AurJ to produce rubrofusarin. Interestingly, AurJ is an O-methyltransferase, and its expression may be inhibited by the accumulation of AdoHcy in this study (Figure 3(e)). The lack of AurJ leads to the accumulation of nor-rubrofusarin and thereby negatively regulates the expression of *PKS12*; it has also been reported that the Δ*FgAurJ* mutant lacks aurofusarin [37,55]. Additionally, both asexual and sexual reproduction were defective in Δ*FgSah1* mutants; the production and germination rate of spores declined and the deletion mutants even produced no perithecia. Furthermore, the pathogenicity of Δ*FgSah1* mutants to coleoptiles and flowering wheat heads were drastically lower than those of PH-1 and Δ*FgSah1-C*, which may have resulted from the defective penetration capability of the mutant strains. Some of the defects may partly result from the decreased expression of hydrophobins, which have been reported to be involved in many morphogenetic processes such as hyphal growth, attachment, water–air interface penetration, and plant infection [40,41]. However, in contrast to their reduced pathogenicity, production of the key pathogenic factor DON in Δ*FgSah1* mutants increased dramatically compared to that in PH-1 and Δ*FgSah1-C*. Previous studies reported that the accumulation of acetyl-CoA, the basic precursor to DON biosynthesis, will furtherly stimulated DON biosynthesis in *F. graminearum* [29,30]. Under the catalysis of PKS12, one acetyl-CoA and 6 manonyl-CoA units are condensed and folded into nor-rubrofusarin in *F. graminearum* [37]. In this study, deficiency in PKS12 may have led to the accumulation of acetyl-CoA, which can explain the increase in DON synthesis. All these results suggest that FgSah1 participates not only in vegetative growth but also in full virulence and DON production.

Tolerance toward environmental pressures is usually a prerequisite for pathogens to carry out normal physiological processes. Our results demonstrate that absence of FgSah1 greatly increased the sensitivity of Δ*FgSah1* toward osmotic pressure. Moreover, the Δ*FgSah1* mutants had lower tolerance toward membrane stress and oxidative stress, but also showed increased resistance to cell wall stressors compared to PH-1 (Figure 7(a,b)). Previous studies have reported that the effects of excessive oxidation stress can be reduced by the accumulation of pigments in filamentous fungi [56]. We inferred that the increased sensitivity of Δ*FgSah1* to oxidation stress may have resulted from the decreased ability to remove excessive H_2_O_2_. Our results above demonstrate that FgSah1 plays important roles in the response to multiple stresses.

Lastly, FgSah1 is involved in glycerol biosynthesis and lipid metabolism in *F. graminearum*. The HOG pathway regulates glycerol content in most organisms by MAPK Hog1 phosphorylation to maintain osmotic homeostasis [46]. We determined that the accumulation of glycerol in all the tested strains with or without NaCl, and the results showed decreased glycerol in Δ*FgSah1* in comparison to PH-1 and Δ*FgSah1-C*. Correspondingly, phosphorylation levels of Hog1 were also reduced in mutant strains (Figure 8(b)). The reduction of glycerol production in Δ*FgSah1* also explained its increased sensitivity to fludioxonil and iprodione, which have been reported to be related to osmotic pressure [50]. The lack of glycerol in deletion mutants leads to a sharp decrease in the synthesis of LDs, which has been reported to be crucial to full virulence and proper development [57]. We inferred that FgSah1 may be partly responsible for the impaired infection abilities on the host due to the deficient lipid metabolism in mutant strains.

In summary, this work demonstrates that FgSah1 plays an integral role in the growth and metabolism of the filamentous fungus *F. graminearum*. We find that it is required for the methionine cycle, methylation metabolism, fungal development, full virulence, multiple stress responses, lipid metabolism, and fungicide tolerance.

## Supplementary Material

Supplemental Material

## Data Availability

The datasets generated or analyzed during the current study are available from the corresponding author on reasonable request.
