# Supplementary material for: S-adenosyl-L-homocysteine hydrolase FgSah1 is required for fungal development and virulence in Fusarium graminearum
Source: Virulence. 2021 Aug 23;12(1):2171–85. doi: 10.1080/21505594.2021.1965821 (PMC8386609; doi:10.1080/21505594.2021.1965821)
Supplement: Supplemental Material [file KVIR_A_1965821_SM4467.zip › New folder/Table S1.docx]

**Table S1** Primers used in this study

| Primer | Sequence (5’-3’) | Relevant characteristics |
| --- | --- | --- |
| P1 | ACTGGAGGGTATGCTGAA | PCR primers to amplify *FgSAH1* upstream fragment for construction of the gene deletion vector |
| P2 | AGCCAGCCAACAGCTCCCGAATAAAGGGATTGATGAGA |  |
| P3 | CGCAAACCGCCTCTCCCCCCGCAGGGATGGAAGGA | PCR primers to amplify *FgSAH1* downstream fragment for construction of the gene deletion vector |
| P4 | AATGTCGCCCGCAAAGC |  |
| P5 | AGACGCTCAGTGATGGTT | Nested PCR primers to amplify the 3.7kb deletion vector |
| P6 | GGTGCTGTAATGGACGAA |  |
| P9 | GGATGAGTGGCAACGAC | PCR primers for identification of *FgSAH1* deletion transformants at the left junction |
| P10 | GGTACGAATACGGACAGG |  |
| P11 | GCTCTGGCACAATACTCTT | PCR primers for identification of *FgSAH1* deletion transformants at the right junction |
| P12 | AGGCTATCTACCGCACAA |  |
| P7 | GCTCCCGCCCACAAAT | PCR primers for identification of *FgSAH1* deletion mutants at the target gene |
| P8 | CCCTTACCGACATCACC |  |
| *HPH*-F | GGTCGGCATCTACTCTATT | PCR primers for identification of *FgSAH1* deletion mutants at the *HPH* |
| *HPH*-R | TCTACCCAAGCATCCAA |  |
| Prob-F | CCGTATTCGTACCAAGCA | PCR primers to amplify 431 bp probe for southern blotting |
| Prob-R | AGTGAAAGGGAAGAGGAG |  |
| 5615GFP-F | ACTCACTATAGGGCGAATTGGGTACTCAAATTGGTTGATCCATGACCCGTAGT | PCR primers to amplify the native promoter and open-reading frame of *FgSAH1* fragment for construction of the FgSah1-GFP vector |
| 5615GFP-R | CACCACCCCGGTGAACAGCTCCTCGCCCTTGCTCACACCTGGGCTTTGATGTTCTG |  |
| YZGFP-F | AAGCGTGCTACCGATGT | PCR primers for the identification of the in-frame FgSah1 -GFP fusion vector |
| YZGFP-R | CGTGCTGCTTCATGTGGTCG |  |
| *TRI5-*F | GAGTGTTTCATGCATGGCTACGTC | Quantitative real-time PCR primers for analysis of *TRI*5 expression |
| *TRI5-*R | CTGAGCCTCCTTCACATCGTCC |  |
| *TRI6-*F | TATCGAAAATTATATAACCACATC | Quantitative real-time PCR primers for analysis of *TRI*6 expression |
| *TRI6-*R | CTGAGGGCATTCTGAGTAGCGACA |  |
| *PKS12-*F | AATGGCTTCTTGCACATTTCC | Quantitative real-time PCR primers for analysis of *PKS12* expression |
| *PKS12-*R | GCAATCCGATCCATGAACAA |  |
| *AURJ*-F | AAAAAGCAGCCAAGGAGCAT | Quantitative real-time PCR primers for analysis of *AURJ* expression |
| *AURJ*-R | TTCTGATGACACGCTCCCGTA |  |
| *ACTIN*-F | ATCCACGTCACCACTTTCAA | Quantitative real-time PCR primers for analysis of actin gene expression |
| *ACTIN*-R | TGCTTGGAGATCCACATTTG |  |
| FGSG_05066-F | AAACTTGCGTGGAGAGTCTG | Quantitative real-time PCR primers for analysis of FGSG_05066 expression |
| FGSG_05066-R | CCGTATATCCCACATCAAGCC |  |
| FGSG_08613-F | ATGGGTATCATGGCTCTTGG | Quantitative real-time PCR primers for analysis of FGSG_08613 expression |
| FGSG_08613-R | TGTGGTATTCTTGACGAGCG |  |
| FGSG_01764-F | GCAATTCTCAAGGCAACGC | Quantitative real-time PCR primers for analysis of FGSG_01764 expression |
| FGSG_01764-R | CAATGCACATCTGGAAACCG |  |
| FGSG_09066-F | TCTGAGGTCTGTAACGCAAAG | Quantitative real-time PCR primers for analysis of FGSG_09066 expression |
| FGSG_09066-R | CCCTTTCCACCCTTGTAGATG |  |
