# Supplementary figures and images for: S-adenosyl-L-homocysteine hydrolase FgSah1 is required for fungal development and virulence in Fusarium graminearum
Source: Virulence. 2021 Aug 23;12(1):2171–85. doi: 10.1080/21505594.2021.1965821 (PMC8386609; doi:10.1080/21505594.2021.1965821)

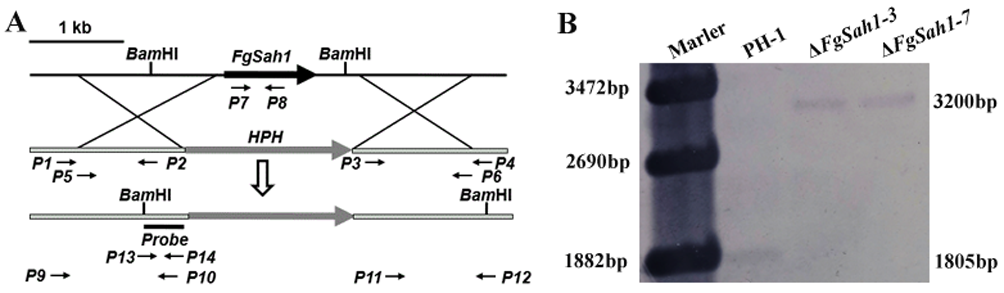

Supplement: Supplemental Material [file KVIR_A_1965821_SM4467.zip › New folder/Figure S1.tif]

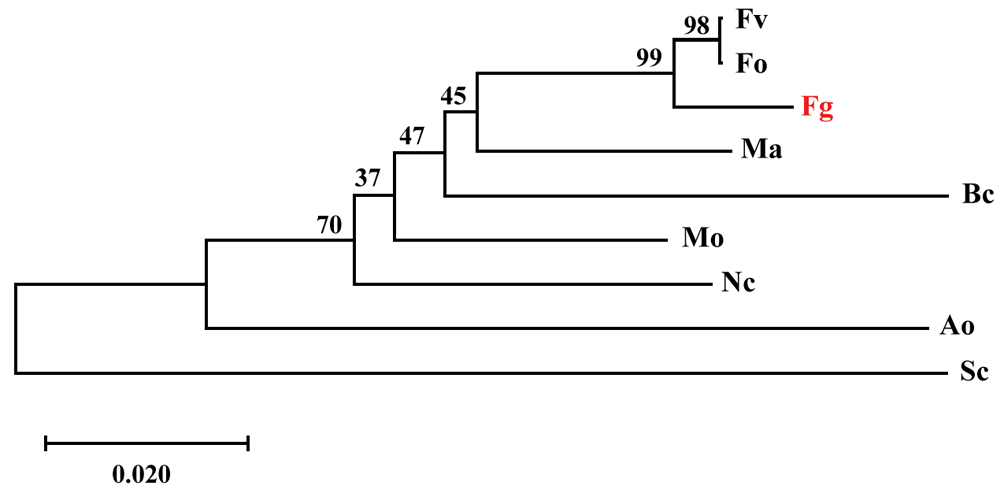

Supplement: Supplemental Material [file KVIR_A_1965821_SM4467.zip › New folder/Figure S2.tif]

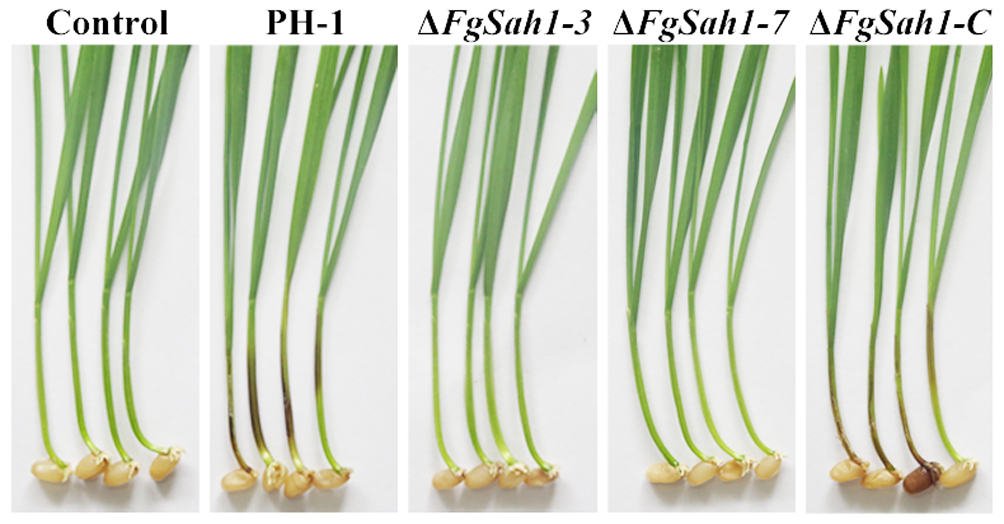

Supplement: Supplemental Material [file KVIR_A_1965821_SM4467.zip › New folder/Figure S3.tif]
